# Pre-pregnancy obesity is associated with an altered maternal metabolome and reduced Flt3L expression in preterm birth

**DOI:** 10.1038/s41598-024-81194-4

**Published:** 2024-12-03

**Authors:** Ismail Sebina, Charles Bidgood, Felicity Stalley, Gunter Hartel, Terra Stark, Leonie Callaway, Akwasi Amoako, Christoph Lehner, Marloes Dekker Nitert, Simon Phipps

**Affiliations:** 1 Infection and Inflammation Program, QIMR Berghofer Medical Research Institute, Herston, QLD 4006 Australia; 2 Faculty of Medicine, The University of Queensland, Brisbane, QLD 4072 Australia; 3 School of Biomedical Sciences, Queensland University of Technology, Brisbane, 4000 QLD Australia; 4 Women’s and Newborn Services, Royal Brisbane and Women’s Hospital, Herston, QLD 4006 Australia; 5 Statistics Unit, QIMR Berghofer Medical Research Institute, Herston, QLD 4006 Australia; 6 School of Public Health, The University of Queensland, Brisbane, QLD Australia; 7 Metabolomics Australia (Queensland Node), The University of Queensland, Brisbane, QLD 4072 Australia; 8 School of Chemistry and Molecular Biosciences, The University of Queensland, Brisbane, QLD 4072 Australia

**Keywords:** Cytokines, Metabolomics, Reproductive biology, Biomarkers, Inflammation, Obesity

## Abstract

Mechanisms linking pre-pregnancy obesity to increased preterm birth risk are unclear. Here, we examined the impact of pre-pregnancy obesity on metabolites, Fms-related tyrosine kinase 3 ligand (Flt3L), and proinflammatory cytokine profiles in preterm birth. We used cytokine bead array, ELISA and Gas Chromatography-Mass Spectrometry (GC-MS) to determine cytokine and metabolite profiles in maternal and cord blood samples from 124 pregnant women in Australia, who gave birth at term (*n* = 86) or preterm (*n* = 38). Besides the expected variations in birth weight and gestational age, all demographic characteristics, including pre-pregnancy body mass index, were similar between the term and preterm birth groups. Mothers in the preterm birth group had reduced Flt3L (*P* = 0.002) and elevated IL-6 (*P* = 0.002) compared with term birthing mothers. Among mothers who gave birth preterm, those with pre-pregnancy obesity had lower Flt3L levels (*P* = 0.02) compared with lean mothers. Flt3L and IL-6 were similar in cord blood across both groups, but TNFα levels (*P* = 0.02) were reduced in preterm newborns. Metabolomic analysis revealed significant shifts in essential metabolites in women with pre-pregnancy obesity, some of which were linked to preterm births. Our findings suggest that maternal pre-pregnancy obesity alters the metabolome and reduces Flt3L expression, potentially increasing risk of preterm birth.

**Supplementary Information:**

The online version contains supplementary material available at 10.1038/s41598-024-81194-4.

## Introduction

Preterm birth, occurring before 37 weeks of gestation, affects nearly 15 million infants annually and poses a significant global health issue^1–3^. It is the primary cause of neonatal mortality and the second leading cause of death (after pneumonia) in children under five^4,5^. Complications from preterm birth impact not only the physical health of mothers and infants but also have significant social, emotional, and economic consequences^6,7^.

A healthy pregnancy requires a balance in the maternal immune system to tolerate the fetus while remaining competent to respond to infections. A failure in this immunological balance can lead to preterm birth^8–10^. The pathophysiology of preterm birth involves a complex interplay of genetic, environmental, social and immunological factors^11^. Obesity in pregnancy increases the risk of adverse pregnancy outcomes, including preterm birth^12–14^. Pre-pregnancy obesity (BMI ≥ 30 kg/m^2^) is linked to spontaneous and iatrogenic preterm birth^15–17^, possibly due to chronic low-grade inflammation and adverse metabolic changes disrupting immune tolerance. Understanding how pre-pregnancy obesity shapes the host-immune landscape in pregnancy is imperative for preventing and/or mitigating its effects on preterm birth in mothers and newborns.

The maternal pregnancy microbiome and its metabolites play essential roles in maintaining the health of both the mother and her unborn child, partially by modulating host immunity and sustaining immune tolerance^18–30^. Dendritic cells (DCs), crucial for effective host immunity and tolerance^31,32^, sustain tolerance at the maternal-fetal interface by inducing regulatory T (Treg) cell and natural killer (NK) cell responses in mice^33–35^. In humans, disruption of this axis is associated with increased risk of pregnancy complications, such as gestational diabetes mellitus (GDM)^36^ and preeclampsia^37,38^, but its impact on preterm birth requires deeper exploration. The Fms-related tyrosine kinase 3 ligand (Flt3L) aids immune regulation^39–41^ by promoting DC development and as a consequence, optimal Treg cell function^41–43^. Our recent study in mice showed that maternal diet-induced microbiome changes during pregnancy disrupt Flt3L expression, impacting DC homeostasis and Treg cell development in the offspring^27^. Consequently, this caused heightened inflammation and significant lung pathology in the offspring during acute respiratory infection. Prior research indicates that exogenous Flt3L administration in abortion-prone mice reduces abortion rates to levels observed in healthy mice^44^, emphasising the importance of the immune system in the maintenance of pregnancy. However, the role of Flt3L in human pregnancy remains unclear, and the relationship between changes in the gestational maternal metabolome and human Flt3L expression in preterm birth has not been explored.

In this study, we used multiplexed cytokine profiling and advanced metabolomic techniques to compare human peripheral and cord plasma samples from mothers who gave birth at term and preterm. We tested the hypothesis that maternal pre-pregnancy obesity predisposes women to preterm birth by altering the circulating metabolome and diminishing Flt3L expression, which may perturb immune tolerance at the maternal-fetal interface.

## Results

### Participant characteristics

For cytokine analysis, we analysed peripheral and cord plasma samples of 86 mothers who gave birth at term (≥ 37 weeks gestation) and 38 mothers who gave birth preterm (Table 1). On average, there were no significant differences in the maternal age, case-matched pre-pregnancy BMI, mode of birth or infant sex between the participants who delivered at term or preterm (Table 1). Rates of gestational diabetes (GDM) and type 2 diabetes were also similar at enrolment and birth in both groups (Table 1). As expected, birth weight, small for gestational age and gestational age at birth were different between the groups (Table 1). There were two (2.3%) infants born small for gestational age (< 10th centile) in the term birth group compared to eight (21.1%) infants in the preterm birth group (Table 1). Rates of preeclampsia were lower among the term birth group (*n* = 18, (21%)) compared to the preterm birth group (*n* = 16, (42%)), (Table 1). For metabolomics analysis, we analysed peripheral and cord plasma samples for a second cohort of 40 mothers who gave birth at term and 36 mothers who birthed preterm. Participants were selected based on matched maternal BMI, maternal age, offspring sex, parity, and mode of birth for metabolic analysis of plasma samples from term and preterm births (Supplementary Table 1).

**Table 1:** Participant characteristics full cohort.

	Preterm birth	Term birth	*P*-value
N	38	86	
Maternal age (years)	30.3 ± 6.8	31.6 ± 5.9	0.28
Prepregnancy BMI (kg/m^2^)	28.8 ± 8.7	27.5 ± 7.9	0.44
BMI category^^^			0.74
≤ 24.9 kg/m^2^ (N (%))	18 (47%)	42 (49%)	
25.0–29.9 kg/m^2^ (N (%))	6 (16%)	18 (21%)	
>= 30.0 kg/m^2^ (N (%))	13 (34%)	25 (30%)	
Gestational diabetes (N (%))	4 (11%)	11 (13%)	0.99
Type 2 diabetes (N (%))	1 (3%)	0 (0%)	
Preeclampsia (N (%))	16 (42%)	18 (21%)	0.01
Parity < 1 (N (%))	21 (55%)	33 (38%)	0.12
Gestational Age at delivery (days)	234 ± 18	272 ± 6	< 0.0001
SGA ((N (%))*	8 (21%)	2 (2%)	0.001
Mode of delivery (CS N(%)/VD N (%))^#^	26 (68%)/10 (26%)	71 (83%) /12 (14%)	0.19

Data is presented as mean ± SD unless otherwise indicated; ND, not determined; CS, Caesarean section; VD, vaginal delivery. ^, data on prepregnancy BMI missing for one mother in the preterm birth group; * data on infant sex and birth centile unknown for 2 infants in the preterm birth group; ^#^ data on mode of delivery unknown for 2 infants in the preterm birth group and 3 infants in the term birth group.

### Reduced Flt3L expression in peripheral blood is associated with preterm birth

**Fig. 1:**
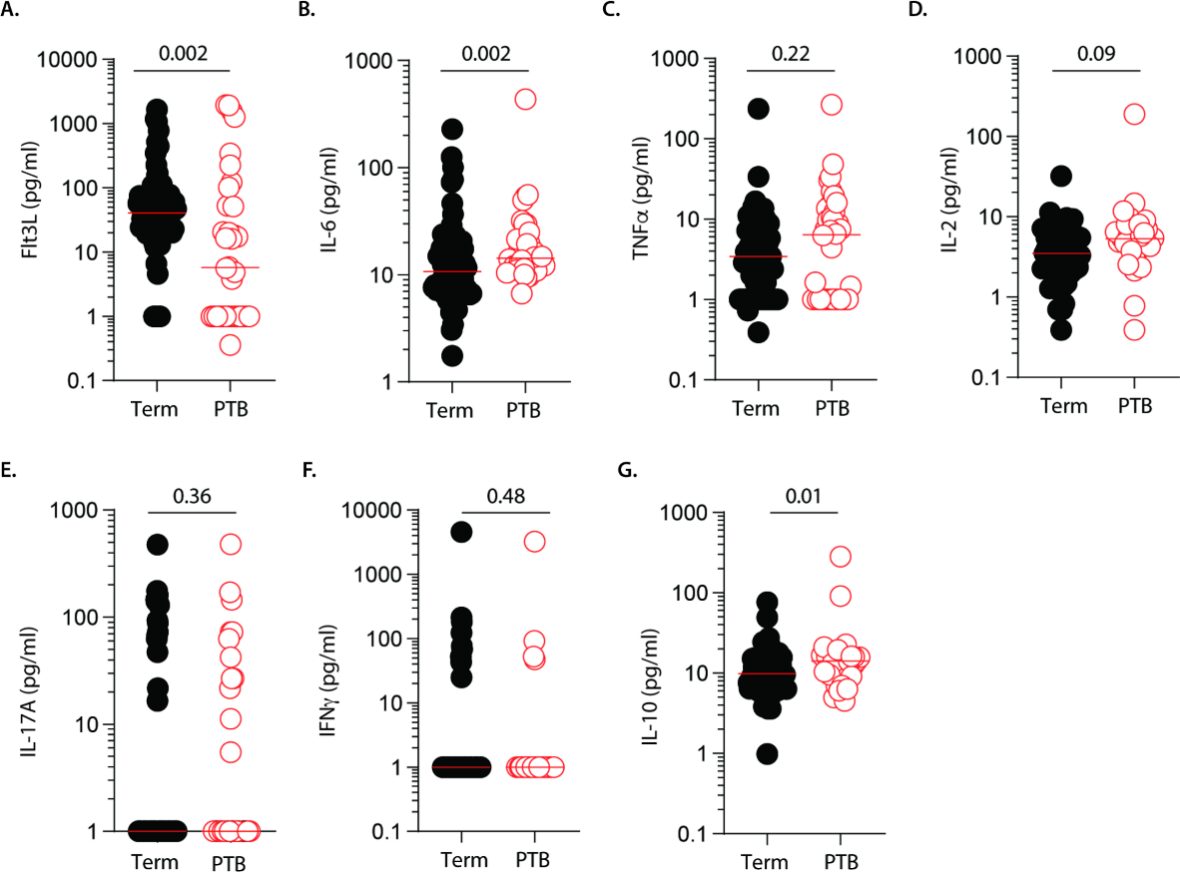
Maternal peripheral blood cytokine expression levels during term and preterm birth. Data depict (**A**) Flt3L, (**B**) IL-6, (**C**) TNFα, (**D**) IL-2, (**E**) IL-17 A, (**F**) IFNγ, and (**G**) IL-10 expression levels in peripheral blood of mothers who delivered at term (*n* = 77) and preterm (*n* = 36). Each dot represents an individual participant and data presented as the median. Statistics: Mann-Whitney U test.

Excessive inflammation in pregnancy increases the risk of preterm birth. To identify cytokines associated with preterm birth, we analysed expression profiles of Flt3L, IL-6, TNFα, IL-2, IL-17 A, IFNγ and IL-10 in peripheral blood (herein: maternal blood; collected prior to delivery) and cord blood samples of mothers who birthed at term (*n* = 86) and preterm (*n* = 38). Cytokine levels varied significantly between the groups; both had samples ranging from very low (or undetectable) to notably higher levels (Fig. 1 and Fig. S1 and S2). In maternal blood, Flt3L expression levels were significantly reduced in plasma samples of mothers who gave birth prematurely compared with those who birthed at term (Fig. 1A). In contrast, elevated levels of the proinflammatory cytokine IL-6 were observed in plasma samples of mothers with preterm *versus* term births (Fig. 1B). Comparable levels of TNFα, IL-2, IL-17 A and IFNγ were observed between both groups (Fig. 1C–F). However, levels of the anti-inflammatory cytokine IL-10 were higher in plasma samples of mothers who birthed prematurely compared with levels detected in mothers who gave birth at term (Fig. 1G). After adjusting for preeclampsia, a potential confounder in preterm birth pathophysiology, our observations remained consistent, with notably reduced Flt3L and increased IL-6, IL-2 and IL-10 levels (Fig. S1). In cord blood, the levels of Flt3L, IL-6, IL-2, IL-17 A, IFNγ and IL-10 were comparable between the groups (Fig. S2A, B and D-G). However, TNFα levels were reduced in cord blood of infants born preterm *versus* those born at term (Fig. S2C). Of note, differences in gestational age had no effect on the cytokine expression levels (data not shown). These data suggest that cytokine profiles in maternal peripheral blood are altered in preterm birth. Specifically, reduced Flt3L expression in maternal peripheral blood, combined with increased IL-6 and possibly elevated TNFα levels are associated with preterm birth, with limited alterations observed in cord blood.

### Pre-pregnancy obesity is associated with reduced Flt3L in preterm birth

**Fig. 2:**
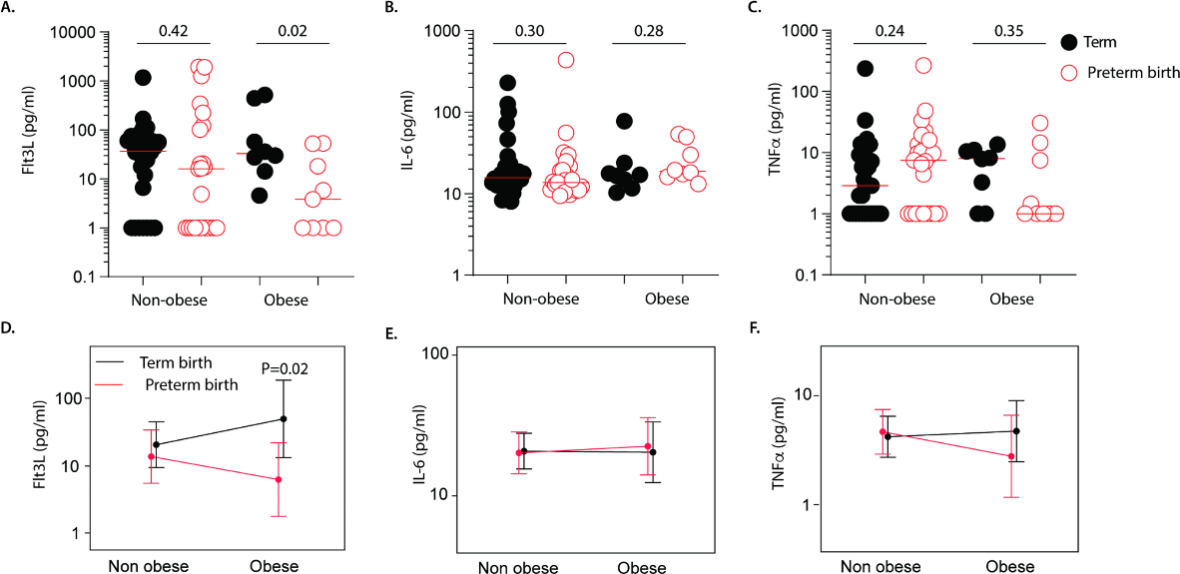
Pre-pregnancy obesity is associated with reduced Flt3L expression in maternal peripheral blood during preterm birth. Data depict (**A**) Flt3L, (**B**) IL-6, (**C**) TNFα expression levels in peripheral blood of mothers without obesity (*n* = 37) and mothers with obesity (*n* = 34) stratified by their timing of birth. (**D**–**F**) depict Two-way ANOVA with interaction analysis of obesity and timing of birth for log-transformed (**D**) Flt3L, (**E**) IL-6 and (**F**) TNFα levels in peripheral maternal blood. Error bars in** D**–**F** represent mean ± SD. Statistics: Mann-Whitney U test (**A**–**C**) and Two-way ANOVA with interaction (**D**–**F**), *P < 0.05.

To determine if pre-pregnancy obesity is linked to the altered Flt3L, IL-6 and TNFα concentrations in pregnancies affected by preterm birth, we compared abundance levels of Flt3L, IL-6 and TNFα in maternal plasma samples of women without obesity (Term; *n* = 30, Preterm; *n* = 25) and women with obesity (Term; *n* = 8, Preterm; *n* = 9) prior to birth (Fig. 2). In mothers without obesity, Flt3L, IL-6 and TNFα expression levels were comparable between those who gave birth at term and preterm (Fig. 2A–C). Intriguingly, Flt3L expression levels were significantly reduced in plasma samples of mothers with obesity who gave birth prematurely compared with those who birthed at term (Fig. 2A), but IL-6 and TNFα levels were similar (Fig. 2B&C). In addition, unlike IL-6 and TNFα, Two-way ANOVA with interaction analysis revealed a strong interaction between low Flt3L expression levels and preterm birth in women with obesity (Fig. 2D–F). These data suggest that women with obesity and reduced Flt3L expression levels in pregnancy are more likely to give birth prematurely.

### Pre-pregnancy obesity alters the maternal metabolome

**Fig. 3:**
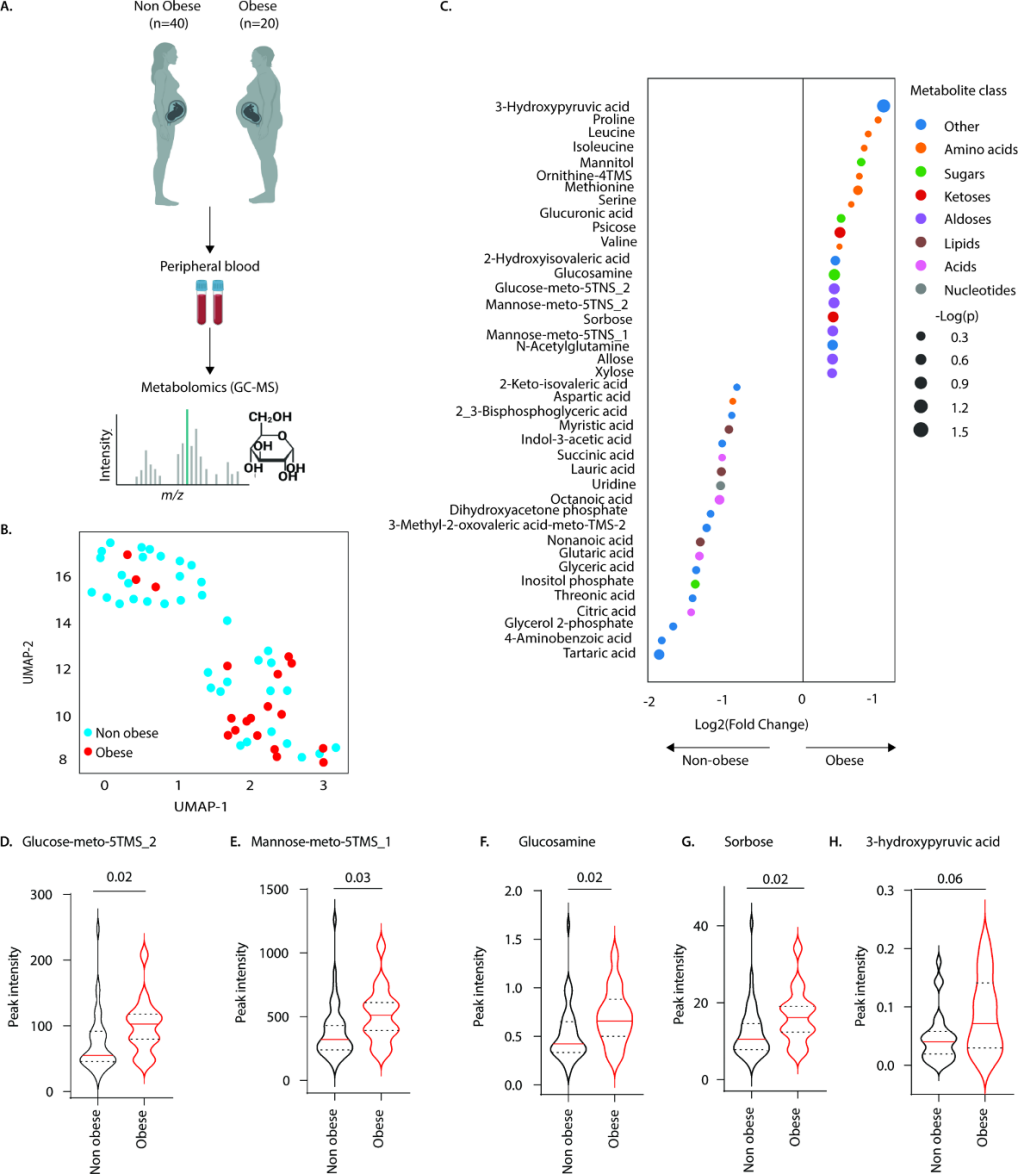
Pre-pregnancy obesity alters the maternal metabolome in pregnancy. GC-MS analysis of metabolite expression levels in peripheral blood of mothers without obesity (*n* = 40) and mothers with obesity (*n* = 20) prior to delivery. (**A**) Study design (**B**) UMAP analysis, and (**C**) differential expression levels of metabolites detected in mothers without obesity and mothers with obesity. Data depict (**D**) glucose-meto-5TMS_2, (**E**) mannose-meto-5TMS_1, (F) glucosamine, (**G**) sorbose, and (**H**) 3-hydroxypyruvic acid levels in peripheral blood of mothers without obesity and mothers with obesity prior to delivery. Statistics: Mann-Whitney U test.

To determine the effects of pre-pregnancy obesity on metabolomic profiles in maternal blood, prior to birth, we next analysed metabolites in plasma samples of women without obesity (*n* = 40) and with obesity (*n* = 20) in pregnancy using gas chromatography-mass spectrometry (GC-MS; Fig. 3A). A total of 135 metabolites were detected (data not shown). The non-linear dimensionality-reduction UMAP (uniform manifold approximation and projection) algorithm revealed a tendency for differential expression patterns of metabolites between the groups (Fig. 3B). We identified alterations in 40 metabolites of which 20 metabolites were upregulated and 20 were downregulated in mothers with obesity (Fig. 3C). The most significantly upregulated metabolites in mothers with obesity included amino acids (proline, leucine, methionine and valine; Fig. 3C), aldoses (glucose-meto-5TMS_2, mannose-meto-5TMS_1, and mannose-meto-5TMS_2; Fig. 3C–E & S3A-B), sugars (mannitol, glucuronic acid and glucosamine (Fig. 3C&F) and ketoses (psicose and sorbose; Fig. 3C&G). Mothers with obesity also exhibited elevated circulating levels of 3-hydroxypyruvic acid compared to mothers without obesity (Fig. 3H). However, tartaric acid, 4-aminobenozic acid, glycerol-2 phosphate, citric acid and threonic acid were downregulated in mothers with obesity prior to birth (Fig. 3C & Fig. S3A). Thus, the maternal metabolome is altered in women with obesity prior to birth.

### Preterm birth is associated with changes in the maternal metabolome

**Fig. 4:**
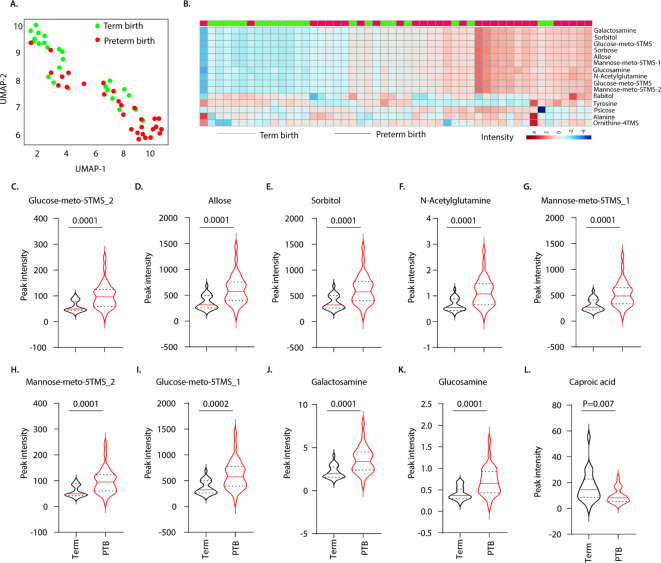
Metabolite expression signatures in maternal peripheral blood during term and preterm birth. GC-MS analysis of metabolite expression levels in peripheral blood of mothers who delivered at term (*n* = 25) and preterm (PTB; *n* = 28). Data depict (**A**) UMAP analysis and (**B**) heatmap analysis of metabolites differentially expressed between mothers who delivered at term and preterm birth. Data depicts (**C**) glucose-meto-5TMS_2, (**D**) allose, (**E**) sorbitol, (**F**) N-acetylglutamine, (**G**) mannose-meto-5TMS_1, (**H**) Mannose-meto-5TMS_2, (**I**) glucose-meto-5TMS_1, (**J**) Galactosamine, (**K**) glucosamine and (**L**) caproic acid expression levels in peripheral blood of mothers who delivered at term and preterm. Statistics: Mann-Whitney U test.

We next determined if alterations in the maternal metabolome at birth are associated with preterm birth. We analysed peripheral blood samples of mothers who gave birth at term (*n* = 25) and preterm (*n* = 28), and a dimensional reduction UMAP analysis revealed differential expression of metabolites between the groups (Fig. 4A). The most differentially expressed metabolites were elevated in mothers who delivered prematurely. These included elevated levels of galactosamine, sorbitol, glucose-meto-5TMS_1, allose, mannose-meto-5TMS_1, glucosamine, N-acetylglutamine, glucose-meto-5TMS_2 and mannose-meto-5TMS_2 (Fig. 4B–K). Higher levels of xylose, galactitol, ribonolactone, psicose and sorbose were also detected in mothers who gave birth preterm *versus* those who birthed at term (Fig. S4A-E). Mothers who delivered at term exhibited increased expression of tyrosine, rabitol and caproic acid compared with mothers who gave birth preterm (Fig. 4B&L). These findings were consistent even after adjusting for preeclampsia in mothers who gave birth preterm (Supplementary Table 2). This indicates that changes in the maternal metabolome are associated with preterm birth.

### Associations between pre-pregnancy obesity and altered metabolites in preterm birth

**Fig. 5:**
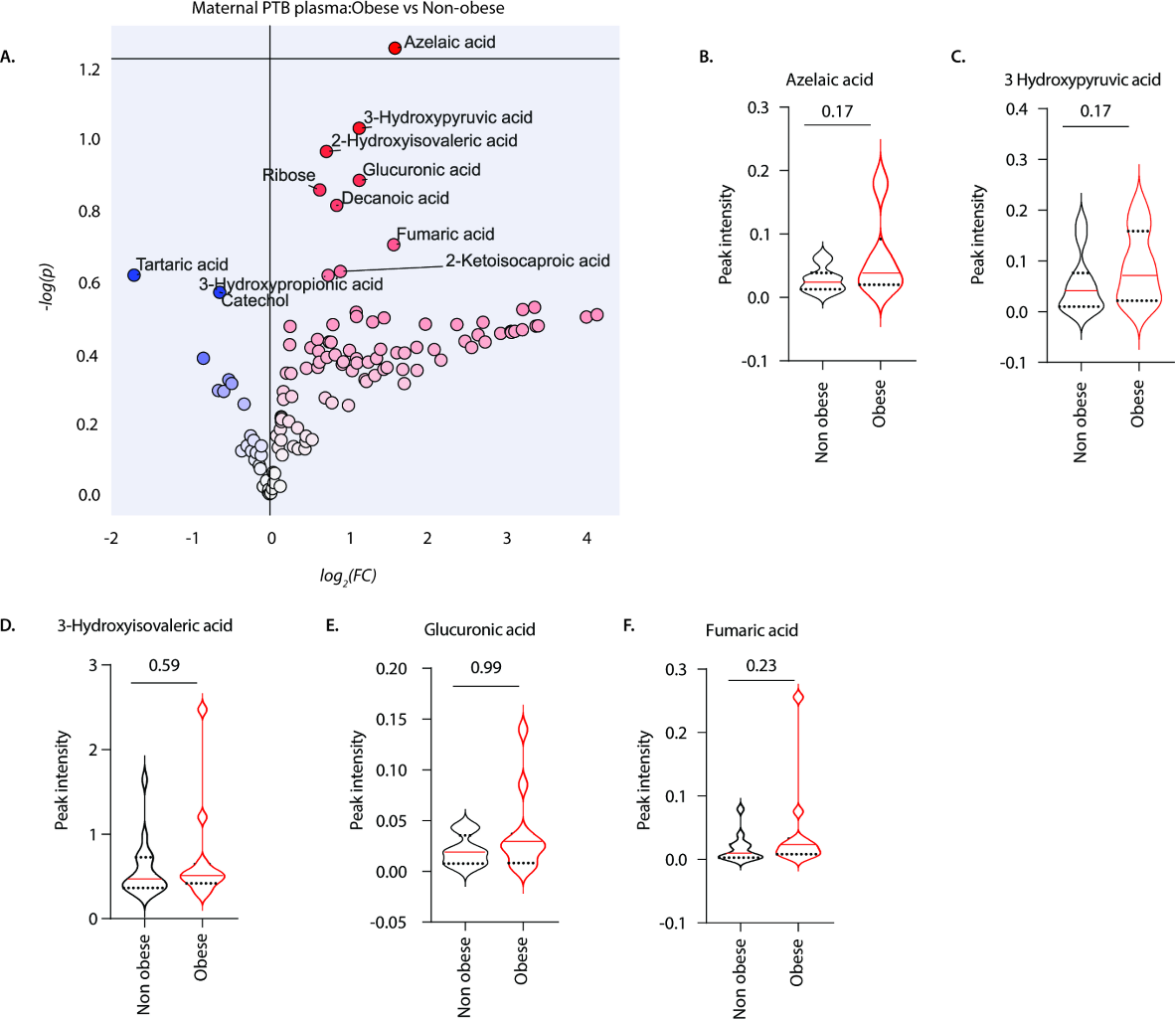
Pre-pregnancy obesity is associated with altered metabolite expression during preterm birth. GC-MS analysis of metabolite expression levels in peripheral blood of mothers without obesity (*n* = 16) and mothers with obesity (*n* = 12) who delivered preterm. Data depict (**A**) volcano plot of metabolites differentially expressed in peripheral blood of mothers without obesity *versus* mothers with obesity. (**B**) depicts azelaic acid, (**C**) 3-hydroxypyruvic acid, (**D**) 3-hydroxyisovaleric acid, (**E**) glucuronic acid and (**F**) fumaric acid expression levels in peripheral blood of mothers without obesity and mothers with obesity who delivered prematurely. Mann Whitney U test.

To determine the effects of pre-pregnancy obesity on metabolomic profiles of mothers who gave birth prematurely, we examined the circulating metabolome in women without (*n* = 16) and with pre-pregnancy obesity (*n* = 12) who delivered preterm (Fig. 5). Based on log2 fold changes, mothers with obesity exhibited a tendency towards higher expression of azelaic acid, 3-hydroxypyruvic acid, 3-hydroxyisovaleric acid, glucuronic and fumaric acid expression compared with mothers without obesity (Fig. 5A). The differences observed overlapped to some extent with the results shown in Fig. 3 when comparing mothers with and without obesity. This was evident for compounds such as 3-hydroxypyruvic acid, 3-hydroxyisovaleric acid, and glucuronic acid. However, the statistical significance of these observations was limited by the small sample sizes analysed (Fig. 5B–F). Nonetheless, these data suggest that pre-pregnancy obesity may alter the maternal metabolome in preterm birth.

### Changes in cord blood metabolome are associated with preterm birth

**Fig. 6:**
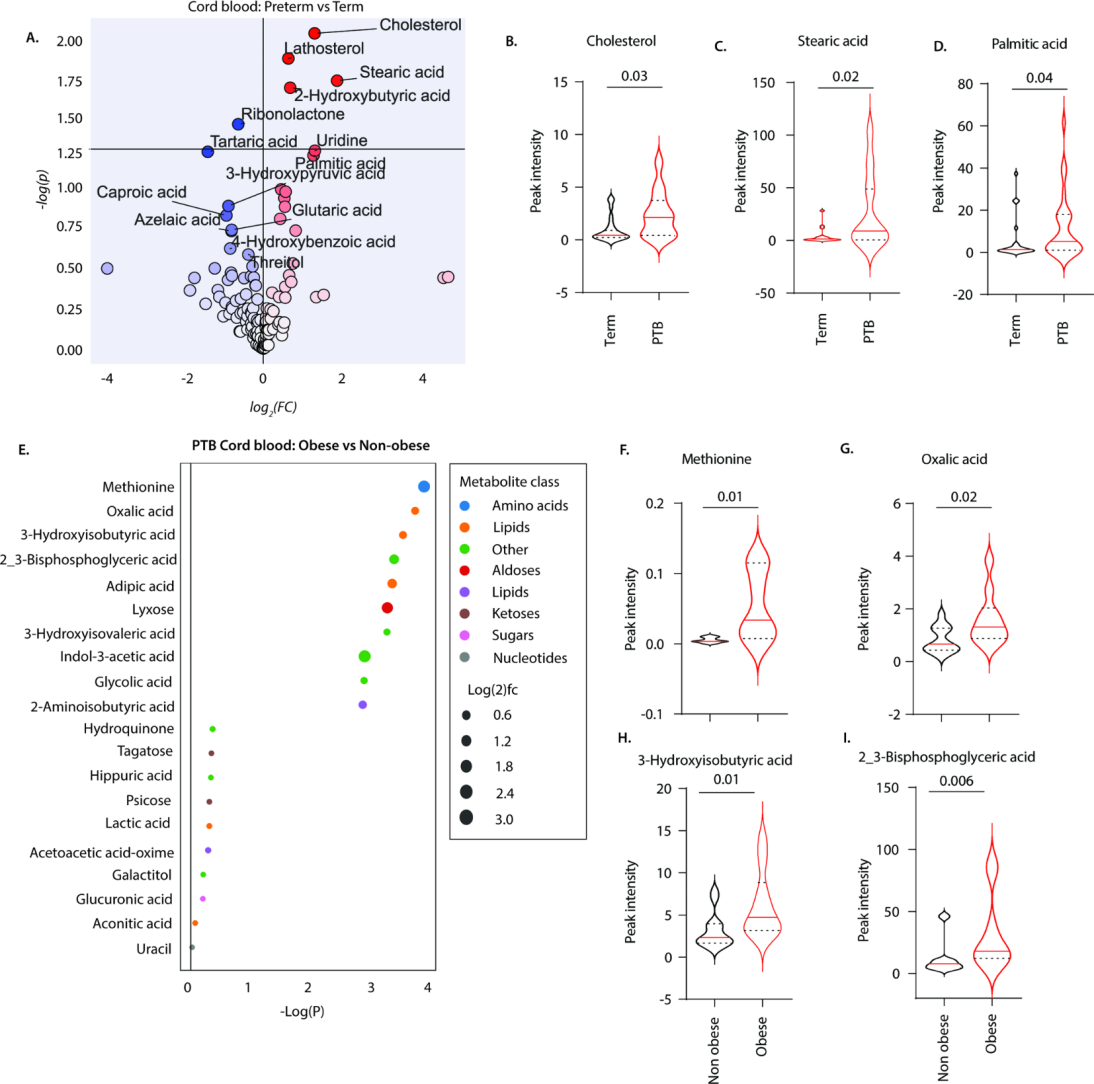
Metabolite expression levels in cord blood during term and preterm birth. GC-MS analysis of metabolite expression levels in cord blood of: (**A**–**D**) mothers who delivered at term (*n* = 20) and preterm (*n* = 23) and (**E**–**F**) mothers without obesity (*n* = 13) and mothers with obesity (*n* = 10) who delivered prematurely. Data depict (**A**) volcano plot of metabolites differentially expressed, (**B**) shows cholesterol, (**C**) stearic acid and (**D**) palmitic acid levels detected in mothers who delivered at term and preterm. Data in (**E**) bubble plot of metabolites differentially expressed between, (**F**) methionine, (**G**) oxalic acid, (**H**) 3-hydroxybutyric acid and (I) 2_3-bisphosphoglyceric acid expression levels in cord blood of mothers without obesity and mothers with obesity who delivered prematurely. Statistics: Mann Whitney U test.

To determine if alterations in the fetal metabolome are also associated with preterm birth, we analysed metabolites in cord plasma samples infants born at term and preterm (Fig. 6). The cord blood of preterm-born infants displayed higher levels of stearic acid, cholesterol, palmitic acid and lathosterol but lower ribonolactone, tartaric acid and caproic acid levels than that of term infants (Fig. 6A–D). To determine if pre-pregnancy obesity is associated with an altered metabolome in preterm born offspring, we compared the levels of different metabolites in cord blood of preterm infants of mothers with obesity and those without obesity. Levels of methionine, oxalic acid, 3-hydroxyisobutyric acid and 2_3-bisphosphoglyceric acid, were increased in cord blood of infants born to mothers with obesity compared to the infants of mothers without obesity (Fig. 6E–I). These data suggest that pre-pregnancy obesity alters the metabolome of the offspring in preterm birth.

## Discussion

In this study, we examined the influence of pre-pregnancy obesity on the cytokine and metabolite profiles in pregnant women delivering at term and preterm. Maternal circulating Flt3L expression was reduced in preterm birth, and this phenotype was more pronounced in mothers who were obese prior to pregnancy. As a hematopoietic growth factor, Flt3L is crucial for immune cell differentiation. In mice, its disrupted expression is linked to imbalances in DC homeostasis and Treg cell development, leading to increased tissue inflammation^27,43^. Moreover, exogenous Flt3L administration in abortion-prone mice reduces abortion rates to levels observed in healthy mice^44^. Thus, the decreased Flt3L levels in mothers who gave birth prematurely may have affected critical immunoregulatory processes during pregnancy. This hypothesis is supported by our observation of elevated pro-inflammatory cytokine IL-6 in the peripheral blood of mothers who gave birth preterm, suggesting increased inflammation during pregnancy, a known risk factor for preterm birth^45^. Interestingly, levels of the anti-inflammatory cytokine IL-10 levels also increased, potentially as a counter-response to the increased inflammation. Moreover, genetic polymorphisms in the IL-10 gene, which are associated with reduced IL-10 production, are linked to increased inflammation and a higher risk of preterm birth^46,47^. However, the precise role of IL-10 in labour remains unclear and challenging to confirm with the current experimental systems. Preterm birth was more frequent in mothers with obesity who expressed lower Flt3L compared to those with higher Flt3L expression levels. However, the intricate relationship between pre-pregnancy obesity, diminished Flt3L, and preterm birth needs further exploration. Our recent studies in mice demonstrated that a poor maternal diet in pregnancy modifies the gut microbiota composition and its metabolites, particularly, short chain fatty acids, which in turn affects Flt3L expression^27^. However, it remains unclear whether diet-induced changes to the maternal microbiota and its metabolites directly influence Flt3L-induced responses associated with preterm birth. In contrast to maternal blood, our cord blood analysis between term and preterm births revealed no differences in cytokine expression levels, except for decreased TNFα in preterm births. This suggests distinct regulatory mechanisms in maternal and fetal compartments, potentially influenced by varying immune environments and developmental stages, or placental barriers. While we identified clear differences in cytokine expression between the term and preterm birth groups, there was significant variability in expression profiles of several cytokines in the maternal and cord blood of both groups. Expression levels varied from barely detectable to significantly elevated. Larger studies are required to validate these findings.

Our study further uncovered associations between pre-pregnancy obesity and shifts in the maternal metabolome before birth. Elevated levels of certain amino acids (proline, leucine, methionine, and valine), aldoses, sugars, ketoses and 3-hydroxypyruvic acid were detected in peripheral blood of mothers with obesity compared with mothers without obesity. In contrast, metabolites such as tartaric acid and threonic acid declined. These changes suggest alterations in protein and glucose intake and metabolism, potentially indicating energy and insulin imbalances. Such metabolic shifts in mothers with obesity could elevate risks for conditions like GDM and preeclampsia^48–50^, particularly as certain amino acids are associated with insulin resistance and oxidative stress. A large study comparing the maternal metabolome of lean women and women with obesity, both with and without GDM, revealed heightened circulating leucine and isoleucine levels in women with obesity, regardless of GDM status^50^. This aligns with our observations, underscoring the increased abundance of branched chain amino acids in women with obesity. However, another report suggested that leucine and isoleucine concentrations were higher in women with obesity, who experienced GDM, suggesting a potential connection between these amino acids and insulin resistance^51^. In this study, our sample size for women with GDM is limited, so we were unable to draw distinct conclusions between the obese and non-obese groups. Nonetheless, our findings highlight the profound associations between obesity and metabolic pathways during pregnancy, which might be linked to adverse pregnancy outcomes. Further studies are essential to understand the precise impact of these metabolic shifts on pregnancy outcomes.

In our study, alterations to the maternal metabolome were also associated with preterm birth. Mothers in the preterm birth group displayed elevated levels of metabolites that mainly are linked to carbohydrate metabolism (e.g. galactosamine, sorbitol, and glucose-meto-5TMS)^52,53^, suggesting potential disruptions in glucose regulation and signalling. In contrast, mothers giving birth at term had increased levels of tyrosine, rabitol, and caproic acid, hinting at variations in protein synthesis and signal transduction (tyrosine), and lipid and carbohydrate metabolism (rabitol/caproic acid) in term and preterm births. Several metabolites associated with lipid metabolism (azelaic acid), glycolysis and gluconeogenesis (3-hydroxypyruvic acid), branched-chain amino acid metabolism (3-hydroxyisovaleric acid), and carbohydrate and citric acid metabolism (glucuronic and fumaric acids) were elevated in maternal blood of women with obesity *versus* women without obesity, who gave birth preterm. However, the statistical significance of these findings is limited due to our study’s small sample size. Larger studies, which also collect detailed dietary intake measurements, are required to validate these findings.

A previous study analysing maternal blood samples from mid-pregnancy (24–28 weeks gestation), identified significant fatty acid metabolite enrichment in the blood of mothers who gave birth preterm^54^. Although we did not observe this in maternal blood, similar patterns emerged in the cord blood of preterm newborns. Elevated fatty acids are linked to increased lipid peroxidation reactions, which can damage tissues of the newborn^55^. Methionine, oxalic acid and 3-hydroxyisobutyric acid were higher in cord blood from infants of obese *versus* non-obese mothers who birthed preterm. In addition, cord blood of preterm newborns from mothers with obesity showed increased levels of stearic acid and cholesterol, and reduced ribonolactone and tartaric acid levels, linked to amino acid and energy metabolism. While these differences could reflect alterations in metabolism that are associated with gestational age, it could also reflect differences in maternal metabolism or metabolic regulation between mothers with obesity and lean mothers or differences in placental metabolism.

Among all the metabolites examined in this study, tartaric acid and 3-hydroxypyruvate consistently exhibited differential abundance. Tartaric acid levels were lower in cord bloods of newborns of mothers with obesity, especially those who gave birth preterm. In contrast, 3-hydroxypyruvate expression was elevated in maternal blood of obese mothers who birthed preterm, but its levels were lowered in cord blood. Both metabolites play pivotal roles in the glyoxylate and dicarboxylate metabolism pathway. Tartaric acid can transition into 3-hydroxypyruvate via oxaloglycolate. The increased 3-hydroxypyruvate levels might be due to its increased conversion from tartaric acid. Moreover, 3-hydroxypyruvate can also be derived from serine breakdown, which was elevated in the blood of mothers with obesity. Possibly, in the infants of mothers with higher circulating serine and 3-hydroxypyruvate levels, the serine instead is broken down into glyoxylate. The glyoxylate can further convert to oxalate, observed at higher levels in preterm infants of mothers with obesity.

A limitation of our study is its small sample size, which could influence the statistical robustness in detecting subtle differences in metabolite and cytokine levels. However, despite this we observed significant metabolic shifts and changes in Flt3L expression in women with pre-pregnancy obesity, some of which were distinctly linked to preterm births. The cross-sectional nature of the study also precluded the establishment of causal relationships between pre-pregnancy obesity, altered maternal metabolome, and preterm birth, and consequently longitudinal studies to validate these findings are warranted. Previous research indicated that the maternal metabolome remains relatively stable across gestation^56^. However, factors like mode of delivery (e.g. Caesarean-section vs. vaginal birth) rather than gestational age could influence the maternal and cord blood metabolome, particularly lipid metabolites. Our maternal samples were obtained between 24 and 48 h prior to the start of labour and are therefore not affected by labour per se but could be affected by differences in fasting states between women delivering vaginally or by Caesarean section. However, our cord blood samples could be affected by mode of birth and while most of our infants were born by Caesarean section, the mode of delivery could impact our findings. The potential interconnection between the immunopathogenesis of preeclampsia and preterm birth may introduce complexity into the interpretation of metabolomic findings in human studies. However, adjusting for this condition did not impact the observed associations between metabolites and preterm birth. Dietary intake may also affect the circulating metabolome and in our study, we have not collected dietary intake data precluding an analysis of its importance. Our results may be confounded by other environmental, genetic, and socioeconomic factors not examined in these studies. Future research addressing this limitation is warranted. Although most preterm birth samples in our study were from iatrogenic preterm births, a few were from spontaneous preterm births as well. Since these two types of preterm birth are driven by distinct underlying mechanisms, our inability to separate these groups introduces heterogeneity, which may obscure specific metabolomic or cytokine signatures unique to each pathway. Larger, well-stratified studies will be needed to better understand the distinct biological processes underlying spontaneous and iatrogenic preterm births, and to validate our findings. The immune cell compartment was not examined in this study. As such, it remains unclear whether reduced Flt3L expression is associated with perturbations of the DC/Treg cell axis in mothers with obesity who gave birth preterm. Future research should address this limitation.

In conclusion, our study highlights the relationship between pre-pregnancy obesity and preterm birth, with alterations in the maternal metabolome and Flt3L expression at birth. These findings deepen our understanding of the complex interplay between maternal metabolism, immune function, and pregnancy outcomes, and may eventually guide the identification of novel biomarkers or therapeutic interventions for managing preterm birth in vulnerable populations.

## Methods

### Participants

Pregnant women (*N* = 124) between 18 and 45 years of age with a singleton pregnancy were enrolled into the study at the Royal Brisbane and Women’s Hospital, Queensland, Australia. Eighty-six participants delivered at or after 37 weeks of gestational age (Term birth) while 38 participants gave birth prior to 37 weeks of gestation (Preterm birth). The preterm birth cohort included women delivering spontaneously or who were delivered for maternal or fetal reasons. Maternal BMI was assessed at the first antenatal visit, typically within the first trimester. All participants were enrolled with informed consent approvals by the human research ethics committees at the Royal Brisbane and Women’s Hospital (approval numbers HREC/2020/QRBW/64249 and HREC/2008/QRBW/16) and QIMR Berghofer Medical Research Institute (approval number: P3678). All procedures were conducted according to the guidelines of the Australian Code for the Responsible Conduct of Research (2018).

### Sample collection and preparation

Peripheral blood and cord blood samples were collected between 24 and 48 h prior to birth (peripheral blood) or peripartum (cord blood) using VACUETTE EDTA tubes (Greiner Bio-One). Tubes were centrifuged for 10 min at 1600 rpm at 4 °C. Plasma was collected, transferred into screw-cap storage tubes (Thermo Scientific) and stored at − 80 °C until use.

### Quantification of cytokines

The concentration of Flt3L in peripheral and cord plasma samples was analysed by ELISA (R&D systems) according to the manufacturer’s protocol. The concentrations of IL-6, TNFα, IL-2, IFNγ, IL-17 A and IL-10 were quantified in peripheral and cord plasma samples using a multiplexed cytometric bead array (BD Biosciences) as per the manufacturer’s instructions.

### Gas chromatography-mass spectrometry (GC-MS) analysis

Metabolites in human peripheral and cord plasma samples were analysed using GC-MS. Briefly, samples were extracted with 50% methanol containing internal standard U-^13^C-sorbitol (16.6 µM). 35 µL of supernatant was dried in glass-pulled point inserts before being subjected to a two-step derivatization process to increase the volatility and thermal stability of the metabolites. First, samples were mixed with 25 µL methoxyamine hydrochloride in pyridine (30 mg/mL) and incubated at 37 °C for 120 min (min) to protect the carbonyl groups. Next, 25 µL of N, O-Bis(trimethylsilyl)trifluoroacetamide with 1% Trimethylchlorosilane (BSTFA + 1% TMCS) was added, and samples were incubated at 37 °C for 30 min to derivatize acidic protons.

Metabolite profiles were acquired on a Shimadzu GCMS-TQ8050 NX system.

1 µL of derivatised sample was injected into the GC inlet set at 280 °C in split mode of 1:10. Chromatographic separation was achieved using an Agilent DB-5 ms capillary column (30 m × 0.25 mm × 1 μm). Oven conditions were set at 100 °C starting temperature for 4 min, then ramped at 10 °C/min to 320 °C and held for 11 min. Helium was used as the carrier gas at a flow rate of 1 mL/min. Compounds were fragmented by electron impact (EI) ionization and analysed in MRM mode using the Shimadzu Smart Metabolites Database containing 475 MRM metabolite targets.

Data processing was done using Shimadzu LabSolutions Insight GCMS program (v.3.7 SP3). Metabolites were identified by comparing their mass spectra and retention times with those of authenticated standards in the NIST14 mass spectral library and Fiehn GC-MS Metabolomics RTL Library. To account for variability between runs, internal standards were included in each sample, and all samples were randomized and analyzed in triplicate. Data were normalized by dividing each metabolite peak area by the peak area of the internal standard (U-^13^C-sorbitol) and subsequently using the total ion count. Following normalisation of the dataset, dimensional reduction was performed by UMAP using the umap-learn python library^57^. Determination of differential metabolite expression across sample sub-populations (e.g. preterm vs. term and obese vs. non-obese) was carried out by calculation of individual metabolite fold-changes between normalised peak area intensity values for each sub-group shown. Logarithmic transformation of the resultant dataset was performed to identify relative changes in the metabolite expression. Sub-population comparisons were then visualised by volcano plots (Fig. 6A, Sup Fig. 2A) and bubble plots (Figs. 3C and 5A) generated using the seaborn and matplotlib python packages to plot log2(fold change) and negative log-transformed p values determined by a student’s t-test determined using the SciPy python package^58^. Metabolite-specific classes were characterised with guidance from the Human Metabolome Database (HMDB)^59^.

### Statistical analyses

Comparisons between two groups were performed using the Mann-Whitney U-test. Where depicted, Two-way ANOVA and Sidak’s test were used for multiple comparisons among groups. Where depicted, the Kruskal-Wallis One-way ANOVA and Dunn’s test were also employed for multiple comparisons among three or more groups. *P* < 0.05 was considered significant (*p* < 0.05 =*; *p* < 0.01 =**; *p* < 0.001 =***). All statistical analyses were performed using GraphPad Prism v9.5 software (La Jolla, CA).

## Electronic supplementary material

Below is the link to the electronic supplementary material.

Supplementary Material 1

## Data Availability

To protect participant confidentiality, supporting data cannot be made openly available. Bonafide researchers can apply for access through the corresponding author Dr Ismail Sebina (i.sebina@uq.edu.au) or Dr Marloes Dekker (m.dekker@uq.edu.au). Raw data for the metabolomics analysis is available via the Shimadzu Smart Metabolites Database https://www.shimadzu.com/an/products/gas-chromatograph-mass-spectrometry/gc-ms-software/smart-metabolites-database/index.html.
