# Supplementary material for: Pre-pregnancy obesity is associated with an altered maternal metabolome and reduced Flt3L expression in preterm birth
Source: Sci Rep. 2024 Dec 3;14:30027. doi: 10.1038/s41598-024-81194-4 (PMC11615298; doi:10.1038/s41598-024-81194-4)
Supplement: Supplementary file 1 — Supplementary Material 1 [file 41598_2024_81194_MOESM1_ESM.pdf]

Pre-pregnancy obesity is associated with an altered maternal metabolome and reduced Flt3L expression in preterm birth

Ismail Sebina[1,2,3*], Charles Bidgood[3], Felicity Stalley[4], Gunter Hartel[5,6], Terra Stark[7], Leonie Callaway[2,4], Akwasi Amoako[2,4], Christoph Lehner[2,4], Marloes Dekker Nitert[8#], Simon Phipps[1,2,3#]

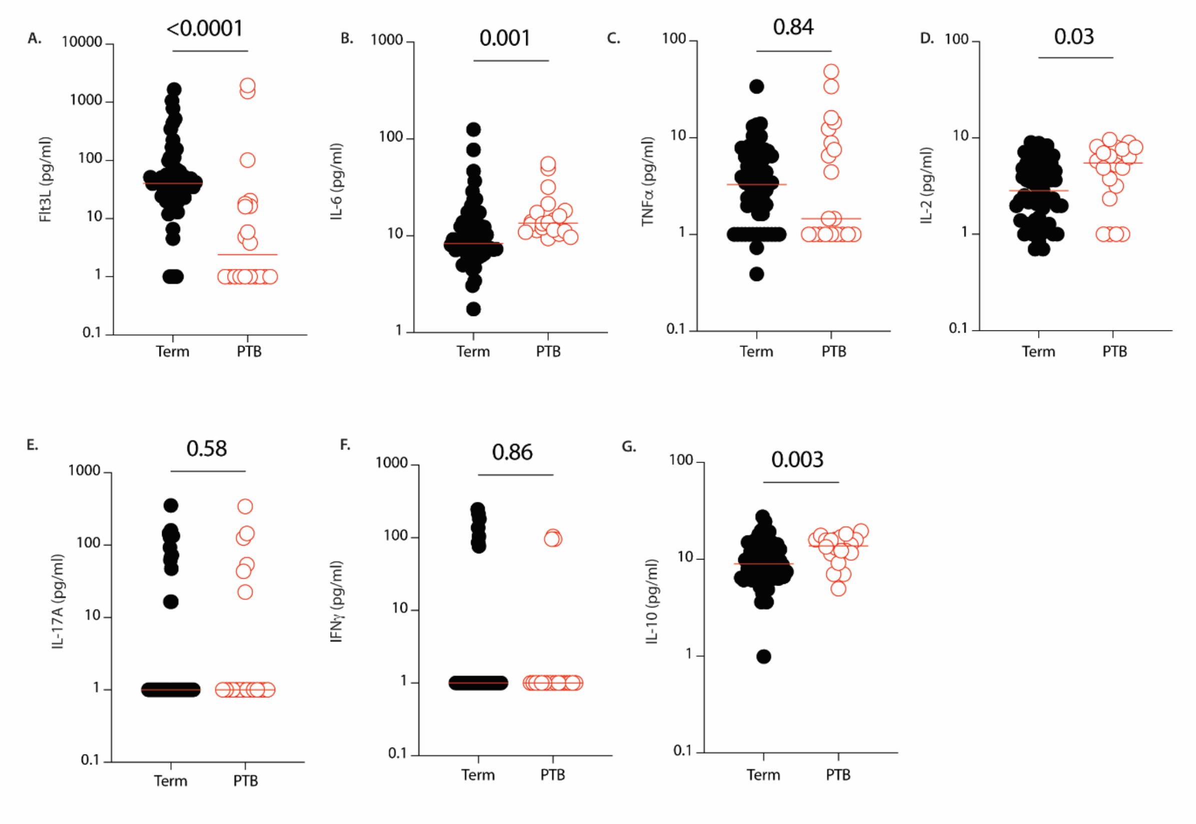

Figure S1. Maternal peripheral blood cytokine expression levels during term and preterm birth adjusted for preeclampsia. Data depict (A) Flt3L, (B) IL-6, (C) TNFα, (D) IL-2, (E) IL-17A, (F) IFNγ, and (G) IL-10 expression levels in peripheral blood of mothers who delivered at term (n=61) and preterm (n=20) after adjusting for preeclampsia. Each dot represents an individual participant and data presented as the median. Statistics: Mann-Whitney U test.

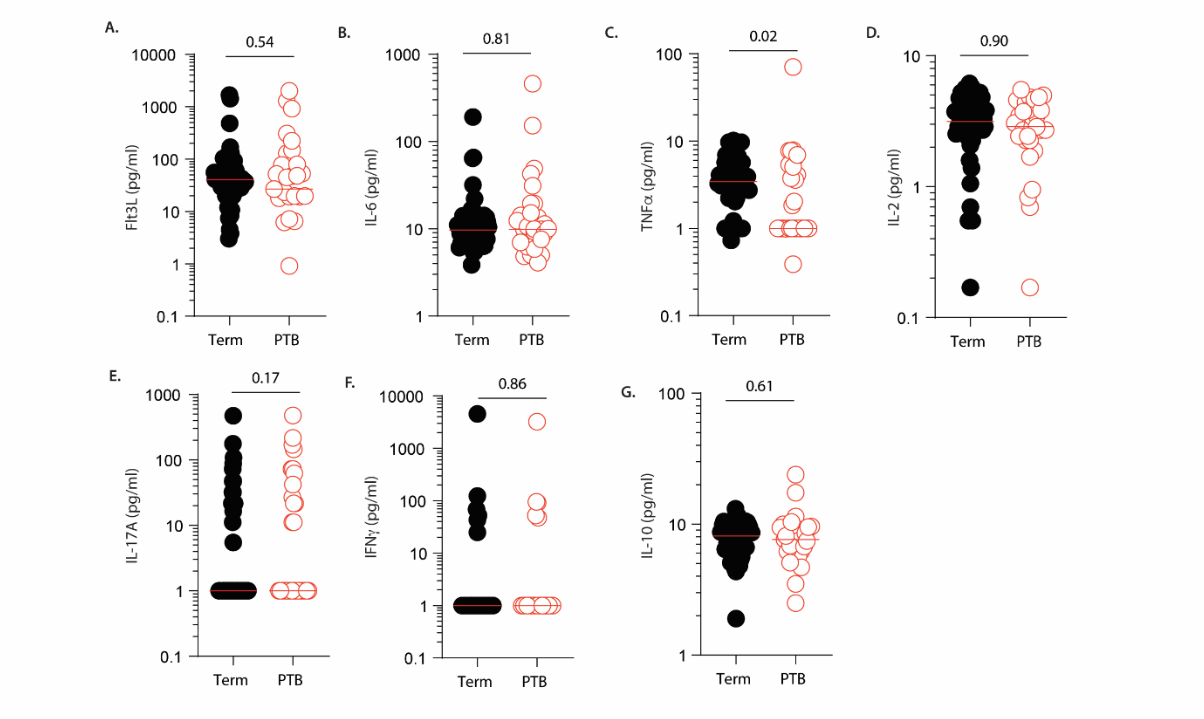

Figure S2. Cytokine expression levels in cord blood during term and preterm birth. Data depicts (A) Flt3L, (B) IL-6, (C) TNFα, (D) IL-2, (E) IL-17A, (F) IFNγ, and (G) IL-10 expression levels in cord blood of mothers who delivered at term (n=82) and preterm birth (n=30). Statistics: Mann-Whitney U test.

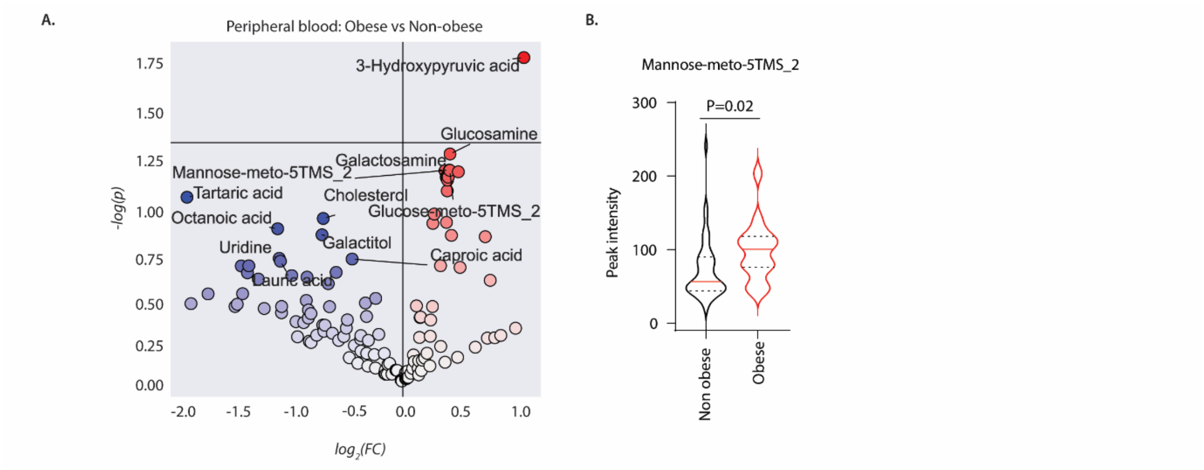

Figure S3. Pre-pregnancy obesity alters the maternal metabolome in pregnancy. GC-MS analysis of metabolite expression levels in peripheral blood of mothers without obesity (n=40) and mothers with obesity (n=20) prior to delivery. Data depict (A) volcano plot of metabolites

differentially expressed and (B) shows mannose-meto-5TMS_2 levels detected in mothers without obesity and mothers with obesity prior to birth. Statistics: Mann-Whitney U test, *P<0.05.

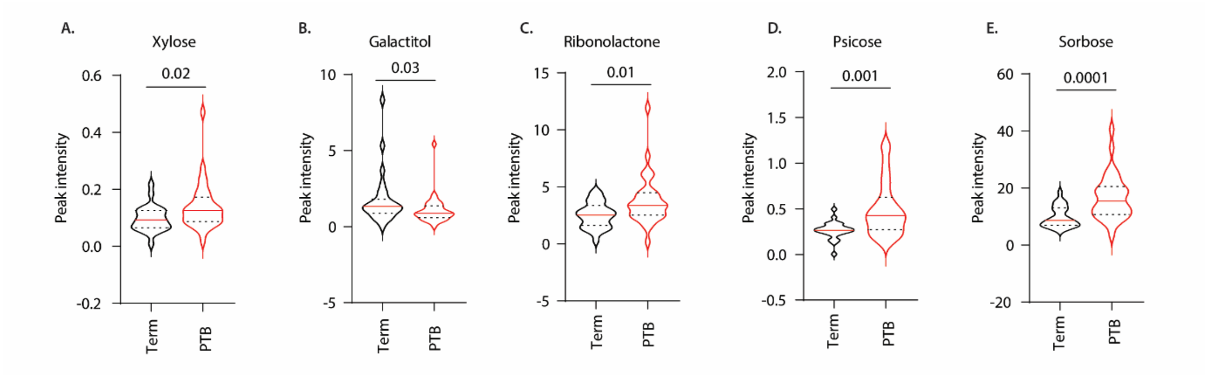

Figure S4. Metabolite expression signatures in maternal peripheral blood during term and preterm birth. GC-MS analysis of metabolite expression levels in peripheral blood of mothers who delivered at term (n=25) and preterm (PTB; n=28). Data depict (A) xylose, (B) galactitol, (C) ribonolactone, (D) psicose and (E) sorbose expression levels in peripheral blood of mothers who delivered at term and preterm. Statistics: Mann-Whitney U test.

Supplementary Table 1. Participant characteristics Metabolomics cohort

	Preterm birth	Term birth	P-value
N	36	40	
Maternal age (years)	30.3 ± 7.0	29.4 ± 6.8	0.58
Prepregnancy BMI (kg/m^2)	28.9 ± 8.9	27.9 ± 9.1	0.61
BMI prior to delivery (kg/m^2)	33.1 ± 9.4	33.0 ± 9.2	0.96
GWG (kg)	12.3 ± 14.4	14.2 ± 6.8	0.47
Booking SBP (mmHg)	119 ± 16	113 ± 13	0.15
Booking DBP (mmHg)	70 ± 12	67 ± 12	0.71
SBP prior to delivery (mmHg)	135 ± 22	128 ± 20	0.08
DBP prior to delivery (mmHg)	83 ± 15	82 ± 14	0.23
Gestational diabetes (N (%))	4 (11.1)	5 (12.5)	0.99
Type 2 diabetes (N (%))	1 (2.8)	0 (0.0)	0.47
Preeclampsia (N (%))	15 (41.7)	16 (40.0)	0.99
Parity < 1 (N (%))	20 (55.6)	21 (52.5)	0.82
Gestational Age at delivery (days)	234 ± 18	273 ± 7	<0.0001
Birth weight (g)	1984 ± 617	3370 ± 435	<0.0001
SGA (N (%))*	7 (19.4)	0 (0.0)	0.005
Infant sex (Female N (%)/Male N (%))*	12 (33.3)/24 (66.7)	20 (50.0)/20 (50.0)	0.17
Mode of delivery (CS N (%)/VD N (%))#	24 (66.7)/9 (25.0)	25 (62.5)/12 (30.0)	0.79

Data is presented as mean ± SD unless otherwise indicated; DBP, diastolic blood pressure; GWG, gestational weight gain; ND, not determined; SBP, systolic blood pressure; SGA, small for gestational age *e.g.* birth weight < 10th centile for gestation; * data on infant sex and birth centile unknown for 2 infants in the preterm birth group; # data on mode of delivery unknown for 2 infants in the preterm birth group and 3 infants in the term birth group.

Supplementary Table 2. Maternal metabolic profiles after adjusting for preeclampsia

Full cohort

**Preterm vs Term (without Preeclampsia)**		
Maternal Plasma		
metabolite	fold_change	p_value
Uridine	0.332	0.039
Galactitol	0.527	0.046
Galactosamine	1.419	0.010
Mannose-meto-5TMS_2	1.444	0.010
Glucose-meto-5TMS_1	1.444	0.008
Sorbitol	1.450	0.008
Glucose-meto-5TMS_2	1.460	0.009
N-Acetylglutamine	1.465	0.009
Allose	1.475	0.006
Mannose-meto-5TMS_1	1.475	0.006
Sorbose	1.491	0.005
Glucosamine	1.507	0.003
Psicose	1.767	0.003

Preterm births only

**Preeclampsia vs. Normal (Preterm Births)**		
Maternal Plasma		
metabolite	fold_change	p_value
3-Phosphoglyceric acid	0.088	0.027
Tagatose	1.290	0.026
Galactosamine	1.464	0.031
Glucose-meto-5TMS_1	1.481	0.034
Sorbitol	1.487	0.033
Allose	1.497	0.031
Glucosamine	1.500	0.022
Xylulose	1.510	0.006
Mannose-meto-5TMS_1	1.513	0.031
Sorbose	1.521	0.029
Mannose-meto-5TMS_2	1.535	0.029
Glucose-meto-5TMS_2	1.545	0.027
N-Acetylglutamine	1.553	0.022
